# PREDICTING THE ONSET OF DEMENTIA WITH LONGITUDINAL OBJECTIVE MEASURES OF PHYSICAL FUNCTION

**DOI:** 10.1093/geroni/igae098.4351

**Published:** 2024-12-31

**Authors:** Sydney Schaefer, Daniel Peterson, Marie Ernsth Bravell, Deborah Finkel

**Affiliations:** Arizona State University, Tempe, Arizona, United States; Arizona State University, Arizona State University, Arizona, United States; Jönköping University, Jönköping, Jonkopings Lan, Sweden; University of Southern California, Los Angeles, California, United States

## Abstract

Older adults with dementia have reduced physical function; however, whether physical function decline accelerates prior to dementia onset remains unclear. This study compared changes in three objective physical function measures for up to 26 years in 518 older adults who did or did not develop dementia: Time to complete a three-meter walk (Gait), grip dynamometry (Grip strength), and time to complete five functional tasks (e.g., putting coins into a slot) (Fine motor). Data from three longitudinal Swedish datasets of older adults (OCTO-Twin, GENDER, SATSA) were analyzed. During the studies, 259 participants were diagnosed with dementia (through clinical consensus); only data prior to the age of dementia onset were included. Propensity matching identified 259 participants who did not develop dementia and whose age at last observation matched the age of diagnosis in the dementia group. Linear latent growth curve models captured longitudinal changes in each measure prior to the onset of disability; thus, intercepts reflect an estimate of physical function at dementia onset or the last timepoint in the study. After controlling for sex, the fine motor test declined faster in the dementia onset group, with differences emerging 9 years prior to dementia onset. Gait and grip strength showed no differences between groups in the rate of change. Fine motor decline also occurred at the same rate as cognitive decline (measured with the Mini-mental status exam). These findings suggest that functional fine motor tasks may identify elevated risk for developing dementia, particularly in cases where memory tests may not be reliable.

